# Single classifier vs. ensemble machine learning approaches for mental health prediction

**DOI:** 10.1186/s40708-022-00180-6

**Published:** 2023-01-03

**Authors:** Jetli Chung, Jason Teo

**Affiliations:** 1 Faculty of Computing and Informatics, Universiti Malaysia Sabah, Jalan UMS, 88400 Kota Kinabalu, Sabah Malaysia; 2 Advanced Machine Intelligence Research Group, Faculty of Computing and Informatics, Universiti Malaysia Sabah, Jalan UMS, 88400 Kota Kinabalu, Sabah Malaysia; 3 Evolutionary Computing Laboratory, Faculty of Computing and Informatics, Universiti Malaysia Sabah, Jalan UMS, 88400 Kota Kinabalu, Sabah Malaysia

## Abstract

Early prediction of mental health issues among individuals is paramount for early diagnosis and treatment by mental health professionals. One of the promising approaches to achieving fully automated computer-based approaches for predicting mental health problems is via machine learning. As such, this study aims to empirically evaluate several popular machine learning algorithms in classifying and predicting mental health problems based on a given data set, both from a single classifier approach as well as an ensemble machine learning approach. The data set contains responses to a survey questionnaire that was conducted by Open Sourcing Mental Illness (OSMI). Machine learning algorithms investigated in this study include Logistic Regression, Gradient Boosting, Neural Networks, K-Nearest Neighbours, and Support Vector Machine, as well as an ensemble approach using these algorithms. Comparisons were also made against more recent machine learning approaches, namely Extreme Gradient Boosting and Deep Neural Networks. Overall, Gradient Boosting achieved the highest overall accuracy of 88.80% followed by Neural Networks with 88.00%. This was followed by Extreme Gradient Boosting and Deep Neural Networks at 87.20% and 86.40%, respectively. The ensemble classifier achieved 85.60% while the remaining classifiers achieved between 82.40 and 84.00%. The findings indicate that Gradient Boosting provided the highest classification accuracy for this particular mental health bi-classification prediction task. In general, it was also demonstrated that the prediction results produced by all of the machine learning approaches studied here were able to achieve more than 80% accuracy, thereby indicating a highly promising approach for mental health professionals toward automated clinical diagnosis.

## Introduction

Mental illness is a health problem that significantly affects how a person feels, thinks, behaves, and interacts with other people. Mental illnesses are of different types and degrees of severity. Some of the major types are depression, anxiety, schizophrenia, bipolar mood disorder and personality disorders. Nowadays, advances in scientific and medical fields have produced very effective medical treatments and technology has also made it possible to predict illnesses in their very early stages.

Machine learning is a technique that aims to construct systems that can improve through experience by using advanced statistical and probabilistic techniques. It is believed to be a significantly useful tool to assist in predicting mental health. Generally, there are various machine learning techniques and research that are still ongoing to generate optimal results. Although it is worth noting that there is no single learning algorithm that universally performs best across all domains, it is still pertinent to identify which class of algorithms can perform best for a particular task environment [1].

In a recent study, a machine learning algorithm was developed to predict clinical remission from a 12-week course of citalopram by Chekroud et al. [2]. The data set is collected from 1949 patients that experienced a depression of level 1. 25 variables from the data set were selected to improve the prediction outcome. Then, the gradient boosting method was deployed for the prediction because of its characteristics that combine the weak predictive models when built. The accuracy of 64.6% was obtained by using the gradient boosting method.

Based on the research paper that was conducted by Sumathi and Poorna, the authors have predicted mental health problems among children through various machine learning techniques [3]. The mental health problems that always occur among children are attention problems, academic problems, anxiety, attention deficit hyperactivity disorder and pervasive developmental disorder. The data set is obtained from a clinical psychologist and contains 60 instances in the text document format. Several features and attributes have been selected for the classification and prediction of mental health problems. Several machine learning techniques have been applied for prediction and accuracy. In the experiment, the machine learning technique called Average One-Dependence Estimator (AODE) has recorded 71% in the accuracy. Meanwhile, Neural Networks show the highest accuracy which is 78%. Next is the Logical Analysis Tree (LAT) is recorded accuracy at 70% meanwhile, the multi-class classifier is at 58% accuracy. Another machine learning technique called Radial Basis Function Network (RBFN) has recorded the accuracy at 57%. Furthermore, both K-star and Functional Tree (FT) have been recorded at 42% accuracy. From the experiments, neural networks can perform the best among the algorithms.

A related study was conducted to predict another mental illness which is post-traumatic stress disorder (PTSD), using a support vector machine by Galatzer-Levy et al. [4]. The data set was made up of longitudinal data with 152 subjects gathered during emergency room admission consequent to a traumatic incident. PTSD symptoms were identified by using the latent growth mixture modelling. Then, the result obtained was used for the prediction of PTSD by using a support vector machine. After applying the support vector machine via MATrix LABoratory (MATLAB), the accuracy was shown to be 64.0%.

A research paper by Sau and Bhakta in 2019 shows the prediction of depression and anxiety among seafarers [5]. Seafarers are easily exposed to mental health problems which typically are depression and anxiety. Hence, machine learning technology has been useful in predicting and diagnosing them for early treatments. The authors obtained a data set of 470 seafarers through interviews. Five classifiers which are Categorical Boosting (CatBoost), Random Forest, Logistic Regression, Naive Bayes and Support Vector Machine were chosen on the training data set with tenfold cross-validation. To determine the strength of the machine learning algorithms, the data set with 56 instances are deployed on the trained model. From the result, CatBoost, which is a boosting algorithm performs the best on this training data set with tenfold cross-validation. For the test data set, the CatBoost algorithm has outperformed the other machine learning algorithms with a predictive accuracy of 89.3% and precision of 89.0%. Meanwhile, logistic regression has performed very well with a predictive accuracy of 87.5% and precision of 84.0%.

The study carried out by Resom et al. showed that mental health problems can be predicted by machine learning with audio features [6]. From the results obtained, predictions from text extraction show that XGBoost which is a boosting algorithm is the best performer with a score of 50%. Next, the K-Nearest Neighbours score is 49% in the mean F1 score. Gaussian Processes and Logistic Regression perform reasonably well and can record 48%. Random Forest recorded the mean of the F1 score at 44% followed by the Neural Networks score at 42%. Support Vector Machine scored the lowest mean of F1 score of 49%.

In Young et al., they utilized network analysis and machine learning approaches to identify 48 schizophrenia patients and 24 health controls [7]. The network properties were estimated from the graphs that were rebuilt using probabilistic brain tractography. Subsequently, machine learning was applied to label schizophrenia patients and healthy controls. The performance of the machine learning models was then analysed and evaluated. Based on the results, the highest accuracy was achieved by the Random Forest model with an accuracy of 68.6%, followed by the Multinomial Naive Bayes with an accuracy of 66.9%. Extreme Gradient Boosting (XGBoost) produced an accuracy of 66.3% while Support Vector Machine produced an accuracy of 58.2%. Most of the machine learning models showed encouraging levels of performance in classifying schizophrenia patients and healthy controls.

A recent study was reported by Tate et al. in 2020 in investigating machine learning approaches to predict the mental health problems in children [8]. The authors used 474 predictors extracted from parental reports and registration data. The performance of the model was tested with the area under the receiver operating characteristic curve (AUC). From the obtained results, the authors showed that Random Forest and Support Vector Machine achieved the highest AUC score of 0.754. Other machine learning models tested such as Logistic Regression, XGBoost and Neural Network achieved similar scores of AUC above 0.700.

A screening tool known as EarlyDetect was used by Liu et al. in 2021 to examine mental illness, which is bipolar disorder, in mental health centres by applying machine learning approaches [9]. The data set contains 955 participants that have completed self-report clinical questionnaires and interviews. From the study, the authors managed to obtain an accuracy of 80.6% with a sensitivity of 73.7% and specificity of 87.5% by using the screening tool. Then, they managed to improve the accuracy by 6.9%, and sensitivity by 14.5%, while maintaining specificity by using the fully combined EarlyDetect model.

Previous studies and research surmised that prediction at an early stage can help to prevent and treat illnesses before becoming chronic and lead to significantly more serious issues. Therefore, this paper aims to apply machine algorithms to classify and predict mental health problems from the data set of questionnaires to generate a valuable result to this end. Machine learning algorithms such as Logistic Regression, Gradient Boosting and Neural Networks are empirically and systematically tested to predict and classify mental health problems. Furthermore, machine learning such as K-Nearest Neighbours, Support Vector Machine and ensemble approaches are being introduced to compare with the suggested machine learning algorithms. The majority voting classifier approach has been used for the ensemble approach in this paper. For further exploration, Deep Neural Networks and Extreme Gradient Boosting were also tested and compared in this comparative study. Finally, the performances of the machine learning algorithms will be then analyzed and the major findings summarized. Hopefully, this paper contributes a systematic and comprehensive research analysis for the practitioner to obtain valuable information regarding the mental health field in helping to determine the clinical diagnosis effectively. For instance, the practitioner will be able to gain insights into the performance of machine learning and apply it to a predictable clinical system which could help to determine mental health accurately and precisely.

In this paper, the sections are organized as follows. The Sect. 1 contains the general overview of this research paper and a summary of the related past studies. Next, the Sect. 2 will present the techniques and procedures used to conduct the experiments. The Sect. 3 will discuss the outcome of the conducted experiments. Meanwhile, the Sect. 4 will further explore and analysis about the result of the experiments. Lastly, a Sect. 5 for Conclusion and future work is presented to summarize the research.

## Methods

The open data set “OSMI Mental Health in Tech Survey” is obtained through an online survey conducted by professional experts of Open Sourcing Mental Illness (OSMI) in 2014 [10]. The survey consists of various questions regarding the respondents’ mental health and their opinion on mental health. The original raw data can be accessed on the OSMI website, and the data were covered by a Creative Commons Attribution License which allows free adapting and sharing of the survey results. This survey is aimed to measure the respondents’ attitudes toward mental health in their workplace in the tech industry.

In general, the data set for this project contains many missing, inconsistent and unnecessary values. Hence, data cleaning is necessary to make it suitable for machine learning models to process the data. In this context, the column of comments, state and timestamp and country are removed from the data due to unnecessary values. Then, the columns of the data set are renamed into a short and straightforward label name. It is noticed that the data set contains unique and excessive values, especially in the columns such as gender, self-employed and work interfere. For the gender part, unrelated answers are removed and the genders are categorized into three parts: male, female and others. After that, the missing values in the column of self-employed have been replaced with of “No” answer. Meanwhile, the values in the column of the work interfere that are missing have been replaced with the answer to “Don’t know”.

The next step is transforming the data set into an understandable and readable format for the machine learning models. The function of the label encoder has been applied and encoding the data set into suitable data and features. Besides that, it is found there are no missing values or data after performing the testing. The data set is ready to be used in the application of machine learning models. After performing the feature selection by applying the Extra Trees Classifier to reduce the chance of over-fitting, the features of age, gender, family history, benefits, care options, anonymity, leave and work interference have been selected for the training of the machine learning models. In this case, the family history determines whether the respondents have a family with mental illness. The next category is benefits to show whether the respondents’ employer provided them mental health benefits. The care options feature is asking the respondent whether they know options for the mental health care provided by their employer. The feature of anonymity, in this case, presents the awareness of the respondents whether their companies or employer will protect their privacy and can be trusted if knowing their mental health status. Another feature is called leave which determines the difficulty for the respondents to ask for medical leave for their mental health conditions. Meanwhile, work interference determines that mental health could interfere with their work. The selected features consist of categorical values except for the age which is a numerical value.

This research is conducted to classify and predict binary mental health problems where no is 0, and yes is 1. The training data set containing the value of components is used to determine the suitable class based on the predictor. The predictor variables in this project are family history, care options, gender, age and others. The target variable which is known as treatment has been selected for the training data set to predict mental health problems. In this project, a preliminary experiment is first conducted using a 70–30 splitting where 70% will be used as the training data set, while the remaining 30% will be used as the testing data set.

The performance evaluation is prepared based on the experimental results obtained. Hence, the calculation of the accuracy, sensitivity, specificity and precision of the result in this research will be obtained based on the confusion matrix. The performance of machine learning algorithms is being compared based on the obtained accuracy to determine the best machine learning algorithm for classification and prediction of mental health problems.

Other than that, the full comparative experiment is conducted by introducing repeated k-fold cross-validation. It is noticed that a single run of the k-fold cross-validation may generate a noisy estimation for the performance of the algorithms. Hence, repeated k-fold cross-validation is introduced to improve the performance of the algorithms due to noisy estimation and reduce the variability linked with a single run of the k-fold cross-validation [11]. Moreover, some researchers mentioned that repeating the k-fold cross-validation will help to stabilize the variability of accuracy estimates [11, 12]. Previous studies suggested that a higher number of repeats in cross-validation would be recommended to stabilize the model selection process when being compared to a lower number of repeats [13, 14]. In this experiment, the number of splits value has been set to tenfold as it is the default and popular value among studies [15, 16]. Each tenfold run is repeated 14 times to ensure minimization of stochasticity in the results.

Generally, machine learning models such as Logistic Regression, Gradient Boosting and Neural Networks have been included because they are commonly used to classify data in the medical field. Additionally, K-Nearest Neighbours and Support Vector Machine have been included in this experiment for comparison in the performances. Finally, the Voting Classifier have been included as a representative ensemble approach as well as Deep Neural Networks and Extreme Gradient Boosting representing the more recent machine learning approaches.

Moreover, Gradient Boosting and Neural Networks have been selected for the additional parameter setting. In this case, the parameter for both machine learning models has been tuned to improve the performance in terms of accuracy. The estimators in the Gradient Boosting algorithm have been replaced with the value of 1000. Besides, the learning rate of the algorithm is set to the value of 0.0001. Next, the max depth refers to the maximum depth of the tree that has been changed to the value of 17. The minimum number of samples required to split an internal node in this setting is introduced and set to the value of 10. The minimum number of samples required to be at a leaf node has been set to 5. Meanwhile, the sub-sample is the fraction of samples for fitting the individual base learners and has been set to a value of 0.5. The loss in this setting refers to the loss function to be optimized and has been applied with exponential. For the additional parameter setting of the Neural Networks algorithm, several changes have been performed. For instance, the hidden layer sizes for this setting have been changed to 30, 50, and 13. Next, the learning rate has been set to a constant value.

## Results

Initially, there are six machine learning models were tested in this study, which are Logistic Regression, Gradient Boosting, Neural Networks, K-Nearest Neighbours, Support Vector Machine and Voting Classifier. The results obtained from the first set of experiments with the default settings are summarized and presented below. Subsequently, Deep Neural Networks (DNN) and Extreme Gradient Boosting (XGBoost) have been added for further exploration and comparison against the initial set of classifiers.

**Table 1:** Preliminary results

Machine learning	Accuracy (%)	Precision (%)	Sensitivity (%)	Specificity (%)
Logistic regression	79.63	76.19	85.56	73.82
Gradient boosting	81.22	76.13	90.37	72.25
Neural networks	78.57	73.45	88.77	68.59
K-nearest neighbours	81.22	78.43	85.56	76.96
Support vector machine	80.69	74.15	93.58	68.06
Deep neural networks	79.89	73.62	92.51	67.54
Ensemble approach				
Voting classifier	81.75	75.87	92.51	71.20
Extreme gradient boosting	80.69	75.22	90.91	70.68

Table 1 presents the summary of the performance evaluation for the machine learning algorithms in terms of accuracy, precision, sensitivity and specify [17] during the preliminary experiment with the initial setting.

In this case, accuracy has been used and defined as the sum of true positives and true negatives divided by the total number of predictions. Another metric that is used for performance evaluation is precision which is referred to as the success probability of making a correct positive class classification and computed as the number of true positives divided by the total number of true positives and true negatives. In addition, a sensitivity which is known as the recall is labelled as the percentage of the true positive cases that are correctly classified, thus showing how well the algorithms classified the positive cases. Meanwhile, specificity is defined as the true negative cases that are classified as negative to measure how well the algorithms are in classifying the negative cases.

From the result obtained, Voting Classifier obtains the highest accuracy with a score of 81.75%. It is discovered that Gradient Boosting and K-Nearest Neighbours can achieve the same value of accuracy which is 81.22%. Then, the Support Vector Machine obtains accuracy lower than Gradient Boosting and K-Nearest Neighbours with a percentage of 80.69, followed by Logistic Regression with a score of 79.63%. Neural Networks achieve the lowest accuracy with a percentage of 78.57%.

Moreover, the highest percentage of precision is recorded by K-Nearest Neighbours with a percentage of 78.43%, followed by Logistic Regression with a percentage of 76.19%. Gradient Boosting obtains a higher percentage of precision with a score of 76.13% compared to Voting Classifier and Support Vector Machine recording the precision score of 74.15% and 75.87%, respectively. Next, Neural Networks score the lowest precision with a percentage of 73.45%.

In terms of sensitivity, it is noted that the Support Vector Machine records the highest percentage with a score of 93.58% compared to Voting Classifier with a score of 92.51%. Neural Networks record a lower percentage of 88.77% than Gradient Boosting in the sensitivity score which is 90.37%. On the other hand, Logistic Regression and K-Nearest Neighbours present the lowest and the same percentage of the score in the sensitivity which is 85.56%.

For the specificity, K-Nearest Neighbours obtain the highest percentage score which is 76.96%. Logistic Regression can record a higher specificity score with a percentage of 73.82% than Gradient Boosting which can obtain a percentage of 72.25%. Next, Voting Classifier managed to achieve a percentage of 71.20% in the specificity score. Meanwhile, Neural Networks and Support Vector Machine show a lower percentage compared to the other algorithms which are 68.59% and 68.06%, respectively.

The results will be displayed and examined for the final experiment conducted in this paper. In this experiment, the repeated k-fold cross-validation has been applied in the classification to obtain the average classification. In addition, Gradient Boosting and Neural Networks with additional parameter tuning have been implemented in the classification to improve the accuracy of the prediction.

**Table 2:** Final results

Machine learning	Accuracy (%)	Precision (%)	Sensitivity (%)	Specificity (%)
Logistic regression	84.00	82.86	87.88	79.66
Gradient boosting	88.80	84.21	96.97	79.66
Neural networks	88.00	84.00	95.45	79.66
K-nearest neighbours	84.00	84.85	84.85	83.05
Support vector machine	82.40	84.38	81.82	83.05
Deep neural networks	86.40	80.25	98.47	72.88
Ensemble approach				
Voting classifier	85.60	83.30	90.91	79.66
Extreme gradient boosting	87.20	84.72	92.42	81.36

Table 2 presents the final experiment of machine learning algorithms tested with repeated tenfold cross-validation, the highest accuracy is achieved by the Gradient Boosting algorithm with the additional parameter tuning with a percentage of 88.80% in the final experiment. Next, the Neural Networks algorithm with the additional parameter tuning scores the accuracy with a percentage of 88.00%, followed by the Voting Classifier with a score of 85.60%. The Logistic Regression and K-Nearest Neighbours obtain the same value of accuracy with a percentage of 84.00%. Meanwhile, the Support Vector Machine achieves 82.40% of accuracy which is the lowest accuracy in the final experiment.

In terms of precision, the K-Nearest Neighbours algorithm achieves the highest percentage with a value of 84.85%. Support Vector Machine algorithm obtains a lower precision score than K-Nearest Neighbours with a value of 84.38%. Moreover, the additional parameter tuning for the Gradient Boosting and Neural Networks can obtain precision with the percentage of 84.21% and 84.00%, respectively, which are higher than the Logistic Regression algorithm. Meanwhile, the Logistic Regression algorithm obtains the value of 82.86% in terms of precision.

The Gradient Boosting with additional parameter tuning manages to achieve a remarkable score in sensitivity with a value of 96.97%. Neural Networks with the additional parameter tuning can obtain slightly lower than the Gradient Boosting with additional parameter tuning with a percentage of 95.45%. The Voting Classifier can score a percentage of 90.91% in the sensitivity which is lower than Neural Networks with the additional parameter tuning. Besides that, the Logistic Regression algorithm scores a better percentage of 87.88% than the K-Nearest Neighbours algorithm with a value of 84.85%. The Support Vector Machine algorithm obtains the lowest sensitivity with a percentage of 81.82%.

In terms of specificity, the highest percentage with a value of 83.05% is achieved by the K-Nearest Neighbours algorithm and Support Vector Machine algorithm. Meanwhile, algorithms that are Logistic Regression, Voting Classifier, Gradient Boosting with additional parameter tuning and Neural Networks with additional parameter tuning obtain the same percentage of 79.66% in specificity.

### Deep neural networks and extreme gradient boosting

For further exploration and comparison, DNN and XGBoost have been included in the experiment. The overall results demonstrate that these algorithms performed similarly well when compared to other classifiers conducted in this experiment.

As shown in Table 1, XGBoost achieves a higher percentage of accuracy with a score of 80.69% compared to DNN which is 79.89%. In terms of precision, it is noted that XGBoost obtains a percentage of 75.22%, meanwhile DNN scores the lowest precision among the algorithms which is 73.62%. However, DNN managed to perform slightly better than XGBoost in terms of sensitivity with a percentage of 92.51%. In this case, XGBoost obtains a score of 90.91% in sensitivity. Next, it shows that the DNN obtained the lowest score of specificity which is 67.54%, meanwhile, XGBoost can score a percentage of 70.68% in the specificity.

From Table 2, the final result of the experiment shows that XGBoost managed to increase the accuracy score by a percentage of 87.20%. DNN obtained slightly lower than XGBoost with a score of 86.40% in terms of accuracy. In addition, XGBoost displays the second-highest percentage of precision with a score of 84.72%. DNN scores the lowest score for precision with a percentage of 80.25%. However, DNN presents the highest percentage of sensitivity among the algorithms with a score of 98.47%. Meanwhile, XGBoost scores lower than DNN with a percentage of 92.42%. With a percentage of 81.36% of specificity, it is clearly stated that XGBoost performs well compared to DNN with a score of 72.88%.

## Discussion

**Fig. 1:**
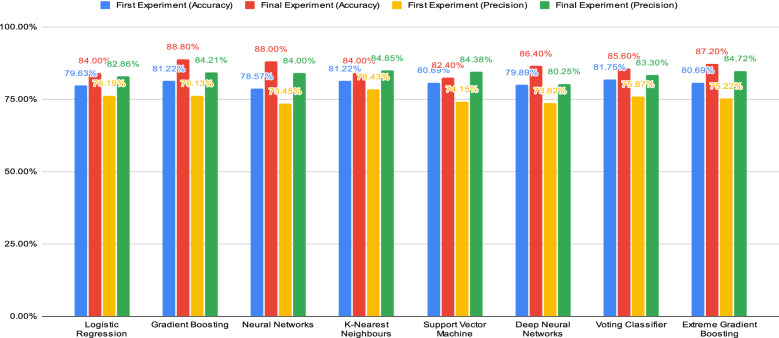
Comparison chart for machine learning algorithms in accuracy and precision

**Fig. 2:**
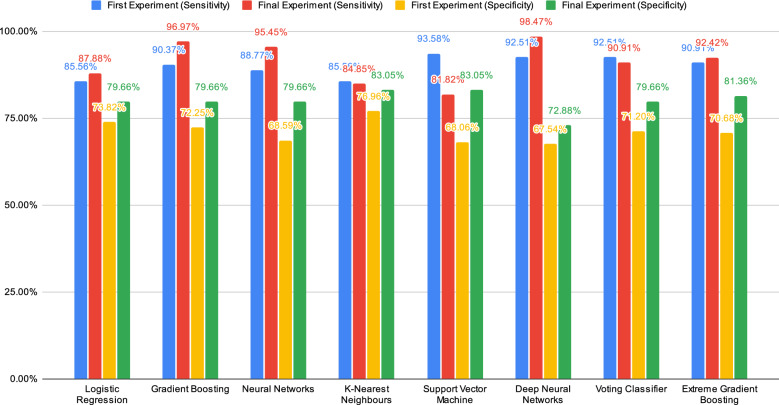
Comparison chart for machine learning algorithms in sensitivity and specificity

Figures 1 and 2 show the comparison of the machine learning algorithms in performances for both preliminary and final experiments. The machine learning algorithms have been compared for both experiments in terms of accuracy, precision, sensitivity and specificity. In this case, the comparison is being conducted to identify and examine the changes in the performance of the machine learning algorithms from both experiments.

The comparison charts show that most of the machine learning algorithms have been improved from the preliminary experiment to the final experiment. The chart shows that Gradient Boosting has the highest accuracy in both experiments. With the additional parameter tuning, Gradient Boosting managed to increase the accuracy score by 7.58%, thus achieving 88.80% in the accuracy score. Meanwhile, Neural Networks show drastic changes in the accuracy score. It shows a great increase in the accuracy from the preliminary experiment to the final experiment by 9.43%. Hence, the percentage accuracy of 88.00% is obtained by Neural Networks with the help of additional parameter tuning which is slightly lower than Gradient Boosting.

In terms of precision, K-Nearest Neighbours have achieved the highest percentage with a score of 84.85% which is increased by 6.42%. However, Gradient Boosting, Neural Networks and Support Vector Machine can present significant changes in terms of precision. Gradient Boosting with additional parameter tuning manages to reach a score of 84.21% in the final experiment from 76.13% in the preliminary experiment. It shows that Gradient Boosting can boost the precision score by 8.08%. Neural Networks with additional parameter tuning show a great impact by increasing the percentage by 10.55%, thus obtaining a score of 84.00% in the final experiment. Meanwhile, the Support Vector Machine manages to obtain 84.38% by increasing the percentage of precision by 10.23% from the preliminary experiment to the final experiment. The Voting Classifier can increase the precision score by 7.43% from the first experiment to the final experiment.

Next, the comparison in terms of sensitivity shows a slight increment in most of the machine learning algorithms except K-Nearest Neighbours and Support Vector Machine. K-Nearest Neighbours have shown a slight decline in the percentage by 0.71%. Meanwhile, Support Vector Machine presents a great decline with a percentage of 11.76%. It is the highest decline where the percentage of sensitivity from 93.58% in the preliminary experiment to 81.82% in the final experiment. Logistic Regression, Gradient Boosting and Neural Networks present an acceptable increase in the percentage. Gradient Boosting can increase the percentage of sensitivity by 6.60% which is from 90.37% to 96.97%. Meanwhile, Neural Networks show a boost in the percentage of sensitivity by 6.68% from the preliminary experiment to the final experiment. It is a great increment where it manages to increase to 95.45% in the final experiment from 88.77% in the preliminary experiment. However, the Voting Classifier shows a small decline of 1.6% in the sensitivity score which is from 92.51% to 90.91%.

In terms of specificity, it can be seen that most of the machine learning algorithms show great changes. From the charts, Gradient Boosting can increase the percentage of specificity by 5.84% from the preliminary experiment to the final experiment. The Neural Networks and Support Vector Machine display a significant increase in the specificity score by 11.07% where the change in increment is higher than changes that occurred in Gradient Boosting. It manages to raise the percentage of specificity from 68.59% to 79.66%. Meanwhile, the Support Vector Machine increases the percentage of specificity by 14.99% which is the highest change recorded. Not only that, the Voting Classifier managed to reach 79.66% of specificity in the final result which is increasing the score by 8.46% from the first result to the final result.

When comparing the single classifiers with the ensemble approach, the experiment conducted shows that the ensemble approach failed to perform better than the Gradient Boosting in terms of accuracy. However, it is able to obtain a good score of 85.60% which is slightly higher than most of the single classifiers such as Logistic Regression, K-Nearest Neighbours and Support Vector Machine. With a percentage of 83.30% in precision, the ensemble approach is being outperformed by most of the single classifiers except for Logistic Regression which is slightly better by a score of 0.44%. In terms of sensitivity, the ensemble approach is unexpectedly higher than Logistic Regression, K-Nearest Neighbours and Support Vector Machine. The result shows that Gradient Boosting and Neural Networks achieve greater scores compared to the ensemble approach which scores 90.91% only in the sensitivity. Moreover, the ensemble approach achieves a score of 79.66% in specificity which is the same degree as most of the single classifiers except K-Nearest Neighbours and Support Vector which is higher by a score of 3.39%. Although the ensemble approach can produce a satisfying performance for predicting mental health problems with higher accuracy and sensitivity, it is still complicated to be explained by the researchers.

### Comparison of deep neural networks and extreme gradient boosting

In this section, the comparison of DNN and XGBoost will be further discussed, including a discussion on the improvement on the prediction results from preliminary experiments to final experiments. Besides that, DNN and XGBoost will be compared to the best classifiers which are Gradient Boosting and Neural Networks. Figures 1 and 2 display the changes that occurred for DNN and XGBoost from the first result to the final result in terms of accuracy, precision, sensitivity and specificity.

In terms of accuracy, it is clearly shown that both algorithms have significant improvements. For instance, DNN managed to increase the accuracy by 6.51% from the first experiment to the final result. Meanwhile, the XGBoost is able to show the same increment with DNN in the accuracy by a percentage of 6.51%.

Next, XGBoost shows a significant boost in precision by a value of 9.5%, thus achieving a percentage of 84.72% in the final result. Even though DNN showed a lower impact in the precision improvement, the classifier managed a percentage of 80.25% which means increased by a value of 6.63%.

When it comes to sensitivity, DNN reached the highest value by increasing the percentage by 5.96% from the first result to the final result. However, XGBoost shows only a minor increment with a value of 1.51%, thus reaching 92.42% in the final experiment.

In terms of specificity, the figures show that XGBoost was able to increase the value of specificity by 10.68%, hence obtaining 81.36% in the final experiment. DNN showed a slight increase when compared to XGBoost. It managed to increase the specificity by a value of 5.34%.

The final results show that Gradient Boosting and Neural Networks managed to perform better than DNN and XGBoost. For instance, the accuracy achieved by Gradient Boosting and Neural Networks is higher than by XGBoost and DNN. In terms of precision, XGBoost appears slightly better than Gradient Boosting and Neural Networks. Meanwhile, DNN showed the lowest percentage of precision. However, the DNN is achieving the highest score in the sensitivity compared to the other algorithms. The final results showed that XGBoost achieves a lower score of sensitivity compared to Gradient Boosting and Neural Networks. When it comes to specificity, XGBoost was able to achieve a better score than Gradient Boosting and Neural Networks. DNN showed the lowest percentage of specificity in the final results.

## Conclusion and future work

From the final results, the Gradient Boosting algorithm with additional parameter tuning has achieved the best performance in terms of accuracy. All the machine learning algorithms experimented with within this research can achieve a satisfying score of accuracy in the classification of mental health problems. However, Neural Networks with the help of additional parameter tuning can achieve drastic and significant improvements in the conducted experiments. Higher accuracy achieved by the machine learning algorithms provides a higher chance of reliability in solving and determining mental health problems. In the additional comparisons utilizing the more recent machine learning approaches of DNN and XGBoost, the experimental results showed that although both were highly promising classifiers for predicting mental health problems, they did not outperform Gradient Boosting and Neural Networks for this particular bi-classification task.

The advancement of machine learning and artificial intelligence technologies presents us with the development of deep learning that maps the input features directly into the outputs through a multi-layer network structure. Thus, it is able to capture the hidden patterns within the data. The deep learning approaches have been very popular in the study of mental health problems. For instance, Mohan and others have applied a deep learning mechanism known as deep feed-forward neural network to obtain information about human brain waves by shaping the raw electroencephalogram signals [18]. From the signals collected, they are able to find that the central regions are insignificantly higher than the other brain regions. The obtained information can be used to differentiate the depressed and normal subjects from the brain wave signals. Moreover, the application of deep learning in neuroimages is also targeted at addressing mental health issues. For predicting depression, Geng et al. have proposed to apply a convolutional neural network and auto-encoder to extract important features from the functional magnetic resonance imaging data [19]. However, the application of deep learning models in mental health issues could encounter several challenges. Firstly, the deep learning technique requires a large volume of data samples to train the models efficiently. This could provide a risk towards several data that are hard to be collected. Besides, collecting massive and different data for training a good deep learning model could be challenging as it needs to consider data redundancy, missing values and deficiency. Not only that, the deep learning model is difficult to interpret and often labelled as a black box. It might become a contentious issue to convince the clinical practitioners about the recommended actions and appropriate procedures generated from the predictive model. Thus, it causes the clinical practitioner to reconsider the mental health prediction through the deep learning model since although it generates good outputs but without clear information about its inner workings.

In general, this research paper has focused on the implementation of machine learning approaches in predicting mental health problems. The empirical testing has shown that the Gradient Boosting algorithm performed best among the individual and ensemble machine learning approaches investigated here, achieving up to 88.8% accuracy. Hence, the result of this study can be useful and helpful for the mental health community, especially in the medical field as an automated computer-based approach to a clinical diagnosis of mental health issues. Researchers and medical practitioners could utilize this achievement in real-world clinical studies where it can become guidance for them to identify or diagnose mental health problems efficiently and effectively. Future investigation to improve prediction performance is planned using Generalized Adversarial Networks (GANs) and transformer neural networks.

## Data Availability

The datasets used and/or analysed during the current study are available from the corresponding author on reasonable request.

## Abbreviations

AUC: Area under the receiver operating characteristic curve
AODE: Average one-dependence estimator
CatBoost: Categorical boosting
DNN: Deep neural networks
FT: Functional tree
GANs: Generalized adversarial networks
LAT: Logical analysis tree
MATLAB: MATrix LABoratory
OSMI: Open sourcing mental illness
PTSD: Post-traumatic stress disorder
RBFN: Radial basis function network
XGBoost: Extreme gradient boosting
